# Method for determining residual total suspended solids retained in pressurized sand filter media

**DOI:** 10.1016/j.mex.2026.103980

**Published:** 2026-06-01

**Authors:** Juliana S. Benítez, Gustavo L. Muniz, Jhonnatan A.Y. Guarnizo, Nicolás D. Cano, Antonio P. Camargo

**Affiliations:** a Agricultural Engineering College, Universidade Estadual de Campinas, Campinas, SP 13083-970, Brazil; b Facultad de Ciencias Agrarias, Universidad Nacional de Colombia, Bogota, Colombia

**Keywords:** Backwashing evaluation, Filter media cleanliness, Microirrigation, Sand bed contamination, Suspended solids extraction

## Abstract

Residual solids retained in the sand bed after backwashing are commonly used to assess the cleaning efficiency of pressurized sand filters employed in irrigation systems. However, procedures originally proposed for slow sand filters are not fully suitable for pressurized filters because these systems differ in operating conditions and bed configuration. In this study, an adapted procedure was developed to determine residual total suspended solids (TSSf) retained in pressurized sand filter media after backwashing. The method involves collecting the upper 0–100 mm layer of the sand bed, followed by drying, homogenization, controlled subsampling, sequential washing cycles under rotational agitation, and determination of suspended solids by vacuum filtration with 0.45 μm glass microfiber filters. Method validation included analysis of grain-size representativeness and reproducibility. The grain-size distribution curves obtained from different subsample masses showed Pearson correlation coefficients higher than 0.99 (p < 0.01), while coefficients of variation for TSSf determination remained below 20% for all evaluated samples. The procedure allows direct evaluation of residual solids retained in pressurized sand filters after backwashing and may be useful for comparing backwashing conditions and assessing filter cleaning performance in irrigation systems.

The main features and applications of this method are as follows:

• Quantification of residual solids retained in pressurized sand filter media after backwashing.
• Representative sand subsampling and extraction of retained solids using sequential washing cycles.
• Evaluation of backwashing performance in irrigation sand filters.

## Graphical abstract

**Figure:**
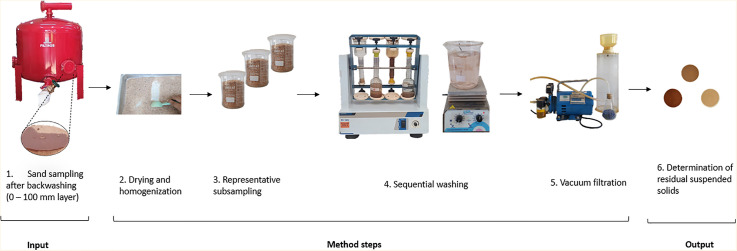

## Specifications table

**Table:** 

**Subject area**	Agricultural and Biological Sciences
**More specific subject area**	Irrigation engineering; filtration systems; pressurized sand filters
**Name of your method**	Residual suspended solids in pressurized sand filter media
**Name and reference of original method**	Total suspended solids determination according to Standard Methods for the Examination of Water and Wastewater (APHA, 2023) [1], adapted from sand filter bed cleanliness assessment methods originally proposed by AWWA (2000) and later modified by Van Staden and Haarhoff (2004a, 2004b, 2006, 2011) [[2], [3], [4], [5]]
**Resource availability**	The method requires standard laboratory equipment, including a drying oven, analytical balance, vacuum filtration system, magnetic stirrer, and glass microfiber filters (0.45 μm)

## Background

Pressurized sand filters play an essential role in localized irrigation systems [6]. Their three-dimensional filtration process allows the effective retention of organic and inorganic particulate matter [7]. Pressurized sand filtration systems operate under two complementary hydraulic modes: filtration and backwashing [8]. During the filtration phase, suspended particles of different sizes are retained within the interstitial voids between sand grains, leading to progressive clogging of the porous medium and a consequent increase in pressure differential across the filter. Once a predefined pressure loss threshold is reached, a backwashing cycle is initiated. Backwashing consists of applying filtered water in reverse flow at sufficient velocity to fluidize the sand bed, promoting hydraulic separation of retained contaminants from the sand grains based on density differences, thereby restoring the filter bed to a condition close to its initial cleanliness [9].

Other sand filtration configurations, such as slow and rapid sand filters used in water treatment plants, have been extensively studied due to their high socio-environmental relevance [10,11]. Consequently, the methodological knowledge developed for these systems has often been extrapolated to pressurized sand filters. However, important differences exist between these technologies, particularly regarding their operating principles, since pressurized sand filters operate in confined systems, with smaller bed volumes and under pressurized flow conditions [12,13].

One of the techniques transferred from water treatment sand filters to pressurized sand filters is the assessment of filter bed cleanliness after backwashing [10]. This approach aims to evaluate backwashing efficiency by collecting sand samples and quantifying the amount of residual contaminants retained in the filter media following an aut-cleaning event. Although conceptually robust, the direct application of this technique to pressurized sand filters is not straightforward due to their distinct hydraulic and geometric characteristics.

The original methodology for assessing residual solids retained in sand beds was first proposed by the American Water Works Association (AWWA) in 2000 and subsequently modified by Van Staden and Haarhoff [[2], [3], [4], [5]] for slow sand filters used in water treatment units. These studies established procedures for sand sampling and laboratory processing to estimate residual total suspended solids retained within the filter media. More recently, this methodological framework has been applied to pressurized sand filters [12,[14], [15], [16]] introducing specific adaptations to sampling and processing steps.

Despite these efforts, a standardized and operational protocol specifically tailored to pressurized sand filters remains lacking. Therefore, this study proposes an adapted method for sand sampling and laboratory processing aimed at quantifying residual total suspended solids retained in pressurized sand filter media, providing a practical and reproducible tool for validating backwashing performance in irrigation filtration systems.

## Method details

### Sand sampling and preparation

Immediately after completion of the backwashing procedure, the upper layer of the filter bed was sampled to determine the residual solids retained in the sand. In pressurized sand filters, the relatively small bed volume and confined geometry allow the complete collection of the selected bed layer, improving the representativeness of the sample compared with conventional water treatment filters.

For each evaluation, all sand contained within the upper 100 mm of the filter bed was carefully removed using a hand scoop and transferred to a clean container. This depth was selected because previous studies have shown that residual contaminants after backwashing are predominantly concentrated near the bed surface [17]. The collected material corresponded to a single composite sample representing the entire sampled layer.

The sand was manually homogenized by thoroughly mixing the material by hand to minimize local variations in particle distribution and contaminant concentration. After homogenization, approximately 1 kg of sand was separated and used as the laboratory sample. The sample was then dried in a forced-air oven at 105 °C for 36 h, or until no further variation in mass was observed, ensuring complete moisture removal and preventing changes in sample composition during storage.

After drying, the sand was allowed to cool to room temperature and was stored in sealed containers until laboratory processing. Drying before subsampling standardized the moisture condition of all samples and reduced variability associated with differences in water content.

### Subsampling of dried sand

**Fig. 1:**
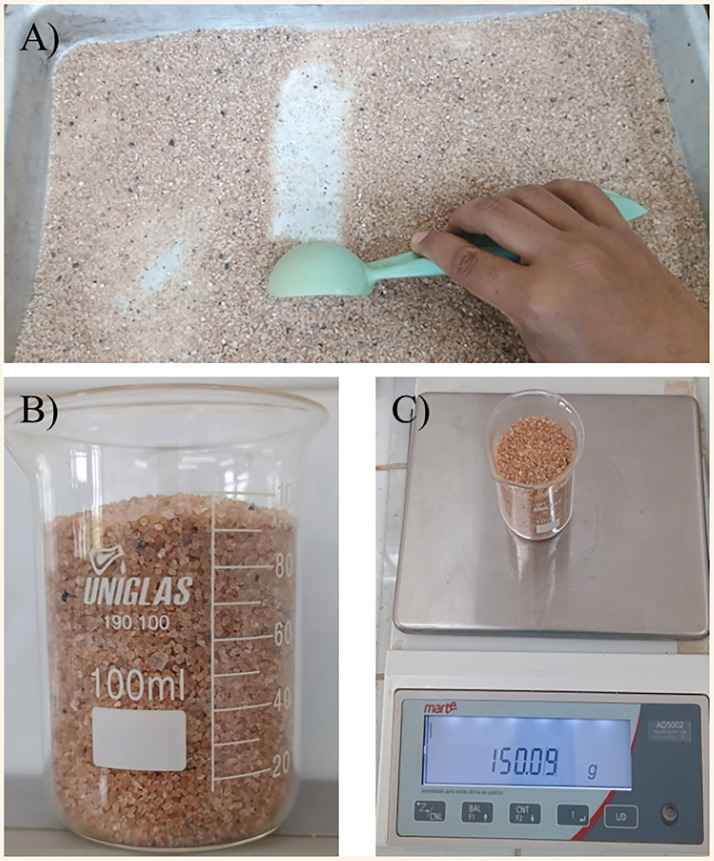
Representative subsampling procedure of dried sand: (A) collection of material along vertical sampling paths across the entire thickness of the spread sand layer; (B) transfer of the collected material to a glass beaker; and (C) weighing and adjustment of the subsample mass to 150.0 ± 0.1 g prior to the extraction of retained solids.

After drying, the sand sample was mixed and evenly distributed on a flat tray, forming a layer of approximately uniform thickness over the entire surface area. The sand was spread in a uniform layer to minimize particle segregation and maintain a representative distribution of both sand particles and retained contaminants. Three independent subsamples of 150 g each were then obtained using a spoon-type spatula. During collection, the spatula was inserted from the surface down to the bottom of the sand layer and moved along vertical sampling paths, allowing the simultaneous collection of particles from different depths of the spread material (Fig. 1A). Collecting material across the entire thickness of the sand layer reduced the likelihood of preferentially sampling either coarse or fine particles.

The collected material was transferred to a 100 mL glass beaker (Fig. 1B), weighed on a precision balance (AD5002, Marte Científica, Brazil) and adjusted to a final mass of 150 ± 0.1 g (Fig. 1C). Three subsamples were analyzed to assess method repeatability while preserving the representativeness of the composite sample.

The subsampling procedure preserved the original grain-size distribution of the filter media and the contaminant particles retained within the bed. Non-representative subsampling may affect the estimation of residual total suspended solids retained in the sand after backwashing.

### Extraction of suspended solids from sand

Each 150 g sand subsample was transferred to a 500 mL glass Stohlmann-type bottle fitted with a sealing cap. Prior to use, all bottles were washed with tap water and visually inspected to ensure the absence of residual particles. Subsequently, 200 mL of distilled water (electrical conductivity < 5 μS cm^−1^) were added to each bottle. Distilled water was used to prevent contamination by external particles during the extraction procedure.

**Fig. 2:**
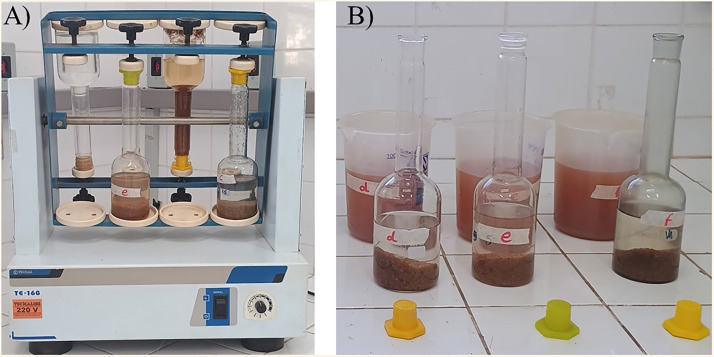
Extraction of suspended solids retained in sand: (A) Wagner-type soil dispersion shaker with 360° rotational motion used to promote repeated washing of the sand subsamples; and (B) 500 mL glass Stohlmann-type bottles containing the water–solid suspension during the extraction procedure.

The bottles were tightly sealed and placed in a Wagner-type soil dispersion shaker (TE-160, Tecnalis, Brazil), which provides a continuous 360° rotational motion (Fig. 2A). The equipment has a capacity for up to eight bottles; however, only three sample bottles were processed simultaneously, while a fourth bottle containing water only was used to maintain mechanical balance during operation. The bottles remained in a vertical position throughout the agitation process.

The shaker rotational speed was adjusted to 15 rpm using the equipment potentiometer. The rotational speed was verified with a stopwatch and adjusted to 15 rpm, corresponding to the manual inversion procedure described by Van Staden and Haarhoff [2]. Under these conditions, the suspension underwent continuous mixing while allowing partial settling of the sand particles during each rotation cycle.

After agitation for 3 min, the bottles were removed from the shaker and the supernatant containing the suspended solids was carefully decanted into a 1 L beaker. Particular care was taken during this step to avoid transferring sand particles together with the liquid phase. No additional settling period was imposed, and the supernatant was transferred immediately after agitation.

Following the first extraction, a new volume of 200 mL of distilled water was added to the sand remaining in each bottle, and the agitation procedure was repeated under the same operating conditions. The supernatants obtained from successive extractions were combined in the same 1 L beaker, resulting in a single composite suspension for each subsample.

A total of five consecutive washing cycles were performed for each subsample, following the recommendations of Van Staden and Haarhoff [5]. Five washing cycles were sufficient to remove particulate material retained between sand grains and provided consistent results among subsamples. At the end of the fifth cycle, the combined suspension was used for the determination of total suspended solids.

When a Wagner-type shaker is not available, the extraction procedure may be performed manually by completely inverting the bottle at least 20 times, allowing partial settling of the sand after each inversion before proceeding with the next cycle.

### Determination of total suspended solids (TSS)

At the end of the five washing cycles, the supernatants obtained from each extraction step were combined in a 1 L beaker, resulting in a composite suspension containing the solids removed from the sand sample. The total volume of the suspension was recorded prior to filtration. To minimize particle settling and ensure homogeneous distribution of suspended solids, the suspension was stirred on a magnetic stirrer at 1500 rpm for at least 1 min immediately before filtration.

Total suspended solids (TSS) were determined by vacuum filtration following the procedures described in the *Standard Methods for the Examination of Water and Wastewater* [1]. Glass microfiber filters (Whatman, nominal pore size of 0.45 μm and diameter of 47 mm) were used for all analyses. Before use, the filters were rinsed with distilled water, dried in a forced-air oven at 105 °C for at least 1 h, cooled in a desiccator to room temperature, and weighed on an analytical balance (ATX224, Shimadzu, Kyoto, Japan; resolution = 0.1 mg) to obtain the initial filter mass (P1, mg).

The entire volume of the suspension (V, L) was then filtered using a vacuum filtration system consisting of a filtration funnel, filter holder, and vacuum pump operating at approximately 40 kPa below atmospheric pressure. Care was taken to ensure complete transfer of the suspension to the filtration unit so that all suspended material removed from the sand sample was retained on the filter surface.

After filtration, the filters containing the retained solids were dried again in a forced-air oven at 105 °C for at least 2 h. Subsequently, the filters were transferred to a desiccator, allowed to cool to room temperature, and weighed on the same analytical balance. The drying and weighing procedures were repeated until constant mass was achieved, and the final filter mass (P2, mg) was recorded.

The concentration of total suspended solids (TSS) in the extracted suspension was calculated from the difference between the final and initial filter masses according to

Eq. (1):

(1) $$TSS=\frac{P2-P1}{V}$$

where TSS is the concentration of total suspended solids (mg L⁻¹), P2 is the final mass of the filter after filtration (mg), P1 is the initial mass of the dry filter (mg), and V is the volume of sample filtered (L).

### Method evaluation

Method evaluation was conducted through two independent tests. The first test aimed to verify the granulometric representativeness of the subsamples, assessing whether the subsampling procedure preserves the original grain-size distribution of the sand bed, regardless of subsample mass. The second test evaluated the reproducibility of the method for estimating residual total suspended solids (*TSS_f_*) retained in the sand bed, using a sand filter prototype operated under controlled laboratory conditions.

For the grain-size distribution analysis, two sand samples with a total mass of 2 kg each were used, obtained from: (i) a sand filter with a clean bed after a backwashing event and (ii) a sand bed artificially contaminated under laboratory conditions. From each bulk sample, subsamples were obtained following the procedure described previously, consisting of three subsamples of 500 g and three subsamples of 150 g for each evaluated condition.

Grain-size analysis was performed by dry sieving using a set of sieves with mesh openings of 2.00 mm, 1.19 mm, 0.71 mm, 0.59 mm, 0.42 mm, and 0.25 mm. Each subsample was mechanically agitated for 10 min, and the percentage of mass retained on each sieve was determined, allowing comparison between subsamples and bulk samples regarding preservation of the sand bed granulometry.

Validation of the method for determining residual total suspended solids retained in the sand was performed using three samples collected from a sand filter prototype operated under laboratory conditions. The filter was subjected to a backwashing event applying an upward velocity of 77.5 ± 1.5 m h⁻^1^ for 1 min.

After backwashing, sand samples were collected and processed according to the protocol described in this study. For each subsample *TSS_f_* were estimated, enabling the assessment of variability among repetitions and, consequently, the evaluation of method reproducibility.

## Method validation

### Grain-size distribution analysis

Table 1 presents the mean values of three replicates of the percentage of material retained on each sieve, for each subsample type and mass analyzed, as well as the overall mean considering all samples. The observed standard deviations were lower than 0.02% for all grain-size classes, indicating high homogeneity in the distribution of the different granulometric fractions composing the subsamples.

**Table 1:** Mean values of three replicates of the percentage of sample retained on each sieve after mechanical agitation.

Sieve opening diameter (mm)	Clean sand subsample		Contaminated sand subsample		Mean retained fraction (%)	Standard deviation
	A1	A2	C1	C2		
	500 g	150 g	500 g	150 g		
2.00	3.8%	3.4%	3.4%	3.2%	3.4%	0.0049
1.19	27.2%	27.2%	26.3%	23.9%	26.1%	0.0211
0.71	67.0%	67.3%	67.7%	70.4%	68.2%	0.0238
0.59	1.5%	1.6%	1.8%	1.8%	1.7%	0.0019
0.42	0.5%	0.6%	0.5%	0.6%	0.5%	0.0008
0.25	0.0%	0.0%	0.2%	0.2%	0.1%	0.0010

**Note:** A1, A2, C1, and C2 correspond to the mean values of three replicates of the percentage of sand particles retained on each sieve. The mean retained fraction represents the overall mean considering all samples, and the standard deviation represents the dispersion around this overall mean.

These results indicate that the subsampling procedure, regardless of the mass used (150 g or 500 g) and the bed condition (clean or contaminated sand), adequately preserves the original grain-size distribution of the filter bed material.

Additionally, Pearson correlation analysis was applied to the grain-size distribution curves obtained for all subsample combinations. The results indicated a high degree of similarity among the curves, with correlation coefficients greater than 0.99 and *p*-values lower than 0.01 in all cases (Table 2). These results confirm that the sampling and subsampling procedures are suitable for preserving the grain-size distribution of the sand bed, ensuring that the subsamples faithfully represent the granulometric condition of the filter media.

**Table 2:** Pearson correlation coefficients between pairs of analyzed grain-size distribution curves.

Comparison	Pearson correlation coefficient	*p*-value
A1 vs A2	0.999	9.9 × 10^−10^
A1 vs C1	0.998	5.9 × 10^−8^
A1 vs C2	0.997	8.0 × 10^−6^
A2 vs C1	0.998	3.1 × 10^−8^
A2 vs C2	0.997	7.0 × 10^−6^
C1 vs C2	0.998	2.2 × 10^−6^

### Analysis of residual total suspended solids in sand

Table 3 presents the mean values of *TSS_f_* retained in the sand along with the corresponding standard deviations and coefficients of variation for each analyzed sample. The variability observed among samples is attributed to subtle differences in the hydraulic conditions of backwashing applied in each test. These variations reflect the inherent heterogeneity of the physical backwashing process and minor fluctuations in application rates, rather than limitations of the laboratory procedure used for *TSSf* determination.

**Table 3:** Residual total suspended solids retained in sand after backwashing, with mean values, standard deviations, and coefficients of variation.

Replicates (subsamples)	Samples		
	A (mg L^−1^)	B (mg L^−1^)	C (mg L^−1^)
R1	16.60	29.08	14.67
R2	17.92	35.33	18.15
R3	19.32	30.14	20.78
Mean	17.9	31.5	17.8
Standard deviation	1.4	3.3	3.1
Coefficient of variation (%)	7.8	10.5	17.3

**Note:** Backwashing rates applied were 77.95 m³ m⁻² h⁻¹ for Sample A, 77.48 m³ m⁻² h⁻¹ for Sample B, and 78.27 m³ m⁻² h⁻¹ for Sample C.

This interpretation is supported by the coefficients of variation lower than 20% observed for all samples, a threshold widely accepted as indicative of good reproducibility in laboratory methods [18,19]. Therefore, the results confirm that the proposed method exhibits good reproducibility for the quantification of *TSSf* in the sand bed of pressurized sand filters.

### Quality assurance and quality control (QA/QC)

All samples were processed using standardized laboratory procedures and identical operating conditions. Sand samples were dried to constant mass prior to analysis, and all determinations were performed using three subsamples under the same washing and filtration conditions. Distilled water and preconditioned glass microfiber filters (0.45 μm) were used throughout the analyses to minimize external contamination and weighing variability. Reproducibility of the method was evaluated using coefficients of variation among replicates, while preservation of the original grain-size distribution after subsampling was verified by granulometric analysis and Pearson correlation coefficients between subsamples.

### Method limitations

The proposed methodology for estimating residual total suspended solids retained in sand presents some limitations that should be considered during sample processing and analysis:

• Sand subsampling must be performed carefully to minimize granulometric distortion. Direct pouring of sand into the sampling container may concentrate larger particles, while direct surface collection from the storage container may produce subsamples that do not adequately represent the filter bed.
• Excessive agitation or agitation with lateral motion reduces method reproducibility and may result in incomplete extraction of suspended solids. For this reason, agitation should be performed using controlled vertical inversion.
• At least five consecutive washing cycles are recommended to release solids retained within the pores between sand particles. Using fewer washing cycles may increase variability among samples and reduce representativeness of the results.

## Ethics statements

This manuscript does not involve human participants, animals, or the use of human or animal data or biological material.

## Data availability

Data supporting the findings of this study are available from the corresponding author upon reasonable request.

## CRediT authorship contribution statement

**Juliana S. Benítez:** Methodology, Investigation, Data curation, Writing – original draft. **Gustavo L. Muniz:** Conceptualization, Methodology, Supervision, Writing – review & editing. **Jhonnatan A.Y. Guarnizo:** Investigation, Formal analysis, Data curation. **Nicolás D. Cano:** Conceptualization, Methodology, Validation, Visualization. **Antonio P. Camargo:** Conceptualization, Supervision, Writing – review & editing.

## Declaration of competing interest

The authors declare that they have no known competing financial interests or personal relationships that could have appeared to influence the work reported in this paper.

## References

[1] APHA (2023).

[2] Van Staden S., Haarhoff J. (2004). A standard test for filter media cleanliness. Water SA.

[3] Van Staden S., Haarhoff J. (2004). Water Quality and Technology Conference and Exposition.

[4] Van Staden S., Haarhoff J. (2006). Proceedings of the Ninth Biennial Conference of the Water Institute of Southern Africa (WISA).

[5] Van Staden S., Haarhoff J. (2011). The use of filter media to determine filter cleanliness. Phys. Chem. Earth.

[6] Arbat G., Pujol T., Puig-Bargués J., Duran-Ros M., Montoro L., Barragán J., Ramírez de Cartagena F. (2013). An experimental and analytical study to analyze hydraulic behavior of nozzle-type underdrains in porous media filters. Agric. Water Manag..

[7] Duran-Ros M., Pujol J., Pujol T., Cufí S., Graciano-Uribe J., Arbat G., Ramírez de Cartagena F., Puig-Bargués J. (2024). Efficiency of backwashing in removing solids from sand Media filters for drip irrigation systems. Agriculture.

[8] Testezlaf R., Deus F.P., Mesquita M. (2014).

[9] de Deus F.P., Alcon G.D., Thebaldi M.S., Diotto A.V. (2025). Effect of the diffuser plate design on the solid removal efficiency of a commercial pressurised sand filter. Biosyst. Eng..

[10] de Souza F.H., Pizzolatti B.S., Sens M.L. (2021). Backwash as a simple operational alternative for small-scale slow sand filters: from conception to the current state of the art. J. Water Process. Eng..

[11] Attiani V., Smidt H., van der Wielen P.W.J.J. (2025). Impact of environmental and process conditions on the microbial ecology and performance of full-scale slow sand filters in drinking water treatment. Water Res..

[12] de Deus F.P., Mesquita M., Salcedo Ramirez J.C., Testezlaf R., de Almeida R.C. (2020). Hydraulic characterisation of the backwash process in sand filters used in micro irrigation. Biosyst. Eng..

[13] Alcon G.D., de Deus F.P., Diotto A.V., Thebaldi M.S., Mesquita M., Nana Y., Zueleta N.A.B. (2023). Influence of the diffuser plate construction design on the filtration hydraulic behaviour in a pressurized sand filter. Biosyst. Eng..

[14] de Deus F.P., Alcon G.D., Thebaldi M.S., Diotto A.V. (2025). Effect of the diffuser plate design on the solid removal efficiency of a commercial pressurised sand filter. Biosyst. Eng..

[15] Duran-Ros M., Pujol J., Pujol T., Cufí S., Arbat G., Ramírez de Cartagena F., Puig-Bargués J. (2023). Solid removal across the bed depth in Media filters for drip irrigation systems. Agriculture.

[16] Graciano-Uribe J., Pujol T., Duran-Ros M., Arbat G., Ramírez de Cartagena F., Puig-Bargués J. (2024). Effects of porous media type and nozzle design on the backwashing regime of pressurised porous media filters. Biosyst. Eng..

[17] Duran-Ros M., Pujol J., Pujol T., Cufí S., Graciano-Uribe J., Arbat G., Ramírez de Cartagena F., Puig-Bargués J. (2024). Efficiency of backwashing in removing solids from sand Media filters for drip irrigation systems. Agriculture.

[18] Reed G.F., Lynn F., Meade B.D. (2002). Use of coefficient of variation in assessing variability of quantitative assays. Clin. Diagn. Lab. Immunol..

[19] Salanon E., Comte B., Centeno D., Durand S., Pujos-Guillot E., Boccard J. (2024). An alternative for the robust assessment of the repeatability and reproducibility of analytical measurements using bivariate dispersion. Chemom. Intell. Lab. Syst..

